# Low concentration press cake protein isolates preserve biological activity during lyophilization and spray drying

**DOI:** 10.3389/fnut.2025.1602010

**Published:** 2025-06-18

**Authors:** Kristina Egger, Lisa Schenzle, Afonso Urich Andreina Isabel, Manuel Zettl, Aleksandra Fuchs, Harald Pichler

**Affiliations:** ^1^acib - Austrian Centre of Industrial Biotechnology, Graz, Austria; ^2^Research Center Pharmaceutical Engineering GmbH, Graz, Austria; ^3^Institute of Molecular Biotechnology Graz University of Technology, NAWI Graz, BioTechMed Graz, Graz, Austria

**Keywords:** HSA alternatives, cultivated meat, press cake proteins, lyophilization, spray drying

## Abstract

A primary challenge in bringing cultivated meat to the market is the high cost of the cell culture media, largely due to their reliance on serum albumins. The production of these albumins is anticipated to become a major bottleneck of this industry. Recently, human serum albumin (HSA) was successfully substituted with seed protein isolates from press cakes enriched with plant albumins. However, these isolates require storage at -80°C to maintain activity, as long-term storage at 4°C or lyophilization leads to aggregation and loss of biological activity. Here, we show that concentrated protein isolates from Styrian oil pumpkin can effectively substitute for human serum albumin (HSA) and support higher proliferation rates in short term experiments as compared to HSA when stored at -80°C (*p* < 0.001), or even when lyophilized (*p* < 0.01). We also demonstrate that protein isolates from Styrian oil pumpkin and rapeseed press cakes perform comparably to HSA (no significant differences) when lyophilized or even spray-dried, provided that the concentration step is omitted. Furthermore, we report protein yields that are four times higher when a more thorough grinding method is utilized. These advancements eliminate the necessity for -80°C storage, thereby facilitating the utilization of locally available press cake protein isolates in media stabilization applications.

## 1 Introduction

Serum albumins, including human serum albumin (HSA) and its fetal variant, fetuin, represent the most abundant proteins in plasma. They serve as the primary carriers of both endogenous and exogenous ligands, such as fatty acids, nucleic acids, hormones, heme, metals, toxins, and drugs. Moreover, they contribute significantly to the pro- and anti-oxidant capacity of plasma and exhibit (pseudo)enzymatic properties (1).

Stabilization of cell culture media with albumins is a broadly applied approach (2), especially in cultivated meat area. Three out of five published serum-free (SF) media compositions contain either human serum albumin (3, 4) or bovine serum albumin with fetuin (5), promoting proliferation of the muscle stem cells. In the context of medium development, albumins act not only as stabilizers of the GFs (6), but also as transport and storage molecules for their ligands, a lot of which are among standard components of the basal cell culture media. In SF cell culture formulations, albumins are used at concentrations of 0.8–5 g/L (3–5), which makes them one of the main contributors to the high culture medium price (7). Also, production of serum albumins is anticipated to become a major bottleneck of cultivated meat industry (8), which rendered looking for alternatives very urgent.

Recently, Stout et al. (9) successfully substituted human serum albumin (HSA) with in-house produced seed protein isolates from press cakes enriched with plant albumins. However, these isolates require considerably high concentration and storage at -80°C to maintain biologic activity, whereas long-term storage at 4°C or lyophilization lead to aggregation and loss of biological activity (9).

In this study, we show that protein isolates from Styrian oil pumpkin (hereafter called pumpkin) and rapeseed press cake can be lyophilized or even spray-dried without significant loss of activity, provided that the concentration step is omitted. We also show that pumpkin protein isolates can replace human serum albumin (HSA) as well as rapeseed protein isolates; in some cases performing even slightly better, depending on the storage method used. Furthermore, we show that the successful preparation of press cake protein isolates is highly dependent on the grinding efficiency of the press cake. These advances eliminate the need for storage at -80°C, which facilitates the use of locally available press cake protein isolates in media stabilization applications.

## 2 Methods

### 2.1 Protein isolation

Five press cake preparations were used to isolate protein fractions - Styrian oil pumpkin (*Cucurbita pepo* var. *styriaca*) protein powder and press cakes of Styrian oil pumpkin, linseed (*Linum usitatissimum*), walnut (*Juglans regia*) and rapeseed (*Brassica napus*). Isolation was preformed following the published protocol by Stout et al. (9) with slight adaptations. Briefly, 10 g press cake preparations were grinded, mixed with 100 mL double distilled water, pH was adjusted to 12.5 using 5 M NaOH and stirred at RT for 1 h. After centrifuging twice for 10 min at 15,000 x g and room temperature (RT), the supernatant was collected without disturbing the pellet and minimally disturbing the fat layer. pH was adjusted to 4.5 using 6 M HCl and the centrifugation step was repeated. Pellets were resuspended in 100 mL double distilled water, pH 12.5. After completely dissolving the proteins, pH was adjusted to 7.2 using 6 M HCl and the centrifugation step was repeated for 30 min. The supernatant was filtered successively though 70 and 40 μm filters and was centrifuged o/n at 21,000 x g and 4°C. The supernatant was then filtered using 0.45 and 0.2 μm filters encountering vehement resistance of the filters. Resulting filtrate was either concentrated using Sartorius Vivaspin Turbo 15, with cut-off 3 kDa (#VS15T92) and then sterile-filtered again, or used directly. Protein concentration of the isolates was determined using Bradford assay, following the protocol of the manufacturer. Protein isolate aliquots were frozen in liquid nitrogen and stored a -80°C.

### 2.2 SDS-page

Thermo-Fisher NuPAGE 4–12% Bis-Tris gels, 15 wells, 1 mm were used in combination with MES Buffer, with Thermo Scientific PageRuler Prestained Protein Ladder (#26616), 5 μL, as a standard. Samples were prepared following manufacturer’s protocol. Gel was run for 35 min at 200 V constant. Instant Blue Coomassie based stain (Sigma-Aldrich # ISB1L) was used to stain all proteins.

### 2.3 Lyophilization and spray drying

The (non-)concentrated protein isolates were stored at -80°C. For processing, protein isolate aliquots were gently thawn in a cooling bath at 4°C.

#### 2.3.1 Lyophilization

For freeze-drying, required quantities of protein isolates were pipetted into a suitable vessel. Then, the vessels were frozen at -20°C for 3 h to freeze the samples, and were subsequently placed in the freeze-dryer at -20°C. The device was initially set to -20°C and was allowed to warm up to -15°C for the main drying phase. This was performed for 20 h at -15°C and 0.045 mbar. For the final drying phase, the temperature was increased to 20°C for four more hours at the same pressure. Lyophilized samples were stored at -20°C until further analysis.

#### 2.3.2 Spray drying

Spray drying was carried out in a Büchi B-290 mini spray dryer following the SOP/manual of the machine. Following settings for spray drying of the protein isolates were used: air (open-loop) was used as drying gas, inlet temperature set to 60°C, aspirator rate at 100% (~35m^3^/h), spray gas flow at ~414 L/h. Nozzle size 0.7 mm was used, spray rate of 30% (~3 g/min from comparable runs, not measured during these experiments), and outlet temperatures set at 40–42°C. The samples were then stored at -20°C until further analysis.

### 2.4 Isolation of satellite cells

Bovine satellite cells (BSC) were isolated from 19 months old Simmental Oxes (*Bos taurus*). Musculus semitendinosus tissue sample was provided by Marcher Fleischwerke GmbH. Cells were isolated from sacrificed animals using a standard protocol described by Stout et al. (10). A ~ 0.5 g of skeletal muscle tissue sample was extracted. The muscle probe was transported in transport medium on ice and immediately further processed, where it was cut up into small pieces and digested in 0.2% collagenase II (Worthington Biochemical #LS004176, Lakewood, NJ, USA; 275 U/mg) for 45–60 min until the paste was homogenized. Digestion reaction was brought to a halt with BSC growth medium (P-GM). The cells were filtered twice, counted using an Invitrogen Countess Automated Cell Counter and plated at a density of 100,000 cells/cm^2^ onto uncoated tissue-culture flasks. Throughout the incubation over 24 h at 37°C with 5% CO_2_ adherent cells attached at the surface of the tissue-culture flask and BSCs stayed in suspension and were transferred to coated flasks at a density of 2000 cells/cm^2^ with 1.5 μg/cm^2^ recombinant human Vitronectin (FisherScientific #15134499). BSCs were left untouched for three to four days before P-GM was changed every two days until a maximum of 70% confluence was reached and the cells were frozen in FBS with 10% dimethyl sulfoxide (DMSO, Sigma #D2650) or passaged for screening or differentiation by using 0.25% trypsin–EDTA (ThermoFisher #25200056).

### 2.5 Confirmation of SCs identity and differentiation

After isolation, undifferentiated SCs were identified by staining for Paired-box 7 (Pax7) marker. SCs were cultured in BSC growth medium (BSC-GM) on a cover glass until they reached 70% confluence, fixed with 4% paraformaldehyde (FisherScientific #AAJ61899AK) for 30 min, washed in DPBS, permeabilized for 15 min with 0.5% Triton-X 100 (Sigma #T8787) in DPBS, blocked for 45 min with 5% goat serum (ThermoFisher #16210064) in DPBS with 0.05% sodium azide (Sigma #S2002), and washed with DPBS and 0.1% Tween-20 (Sigma #P1379). Primary Pax7 rabbit polyclonal antibody (ThermoFisher #PA5-68506) were added at a dilution of 1:500 in blocking solution (Aligent Antibody Diluent #S202230-2) containing 1:100 Alexa Fluor 594 Phalloidin (ThermoFisher #A12381) to cells and incubated at 4°C overnight. Further, cells were washed with DPBS + Tween-20 and incubated with secondary goat anti-rabbit antibody (ThermoFisher #A-11008, 1:500) for 1 h at room temperature, washed with DPBS + Tween-20 and mounted with Fluoroshield mounting medium with DAPI (Abcam #ab104139). Visualization and imaging were performed to validate the satellite cell purity of the isolated cell population, which exceeded 98%.

Isolated SCs were differentiated for ten days. After growing cells to confluency in BSC-GM, the medium was changed to SF Differentiation Medium (DM) (11), and then 50% of the medium was regularly changed (every two to three days) until SCs reached differentiated state and were fixed and prepared as previously described. Phalloidin 594 was diluted 1:100 in blocking solution (Aligent Antibody Diluent #S202230-2) and incubated at 4°C overnight. Fluoroshield mounting medium with DAPI was mounted the day after.

### 2.6 Media composition

Skeletal muscle tissue sample was transferred into transport medium consisting of DMEM + Glutamax (ThermoFisher #10566016) and 1% Antibiotic-Antimycotic (ThermoFisher #15240062) after extraction. For Isolation of BSCs, Primocin growth medium (P-GM) was used containing DMEM + Glutamax (ThermoFisher #10566016), 1% Primocin (Invivogen #ant-pm-1), 20% fetal bovine serum (FBS; ThermoFisher #26140079) and 1 ng/mL human FGF basic/FGF2/bFGF (R&D Systems #233-FB-025/CF). After passage one, P-GM was changed to growth medium (GM) where Primocin is replaced by 1% Antibiotic-Antimycotic (ThermoFisher #15240062). Serum free differentiation medium was prepared according to Messmer et al. (11).

For short-term growth analysis, cells were plated in 96-well (Greiner #655180) containing BSC-GM with 1.5 μg/cm^2^ recombinant human Vitronectin (FisherScientific #15134499). After incubation for 24 h, cells were washed with DPBS (ThermoFisher #14190250) and BSC-GM was changed to screening medium - B8 medium containing DMEM/F12 (ThermoFisher #11320033), HiDef-B8 medium aliquots (Defined Bioscience #LSS-201) and 1% Antibiotic-Antimycotic, supplemented or not supplemented with 0.8 g/L HSA. According to the screening method, defined concentrations of medium components were added.

### 2.7 Short term proliferation experiments with BSCs

BSCs were thawn in 5 mL GM and centrifuged at 200 x g for 3 min. The supernatant was discarded, and the cells were washed with 5 mL DPBS. After another centrifugation step and removal of the supernatant, the cells were carefully resuspended in 5 mL BSC-GM and counted with Invitrogen Countess Automated Cell Counter.

For short term analysis SCs (800 cells/well = approx. 2000 cells/cm^2^) were plated in 96-well tissue culture plates (Greiner #655180) containing 100 μL BSC-GM with 1.5 μg recombinant human Vitronectin (FisherScientific #15134499)/cm^2^. After 24 h of incubation at 37°C and 5% CO_2_, the cells were rinsed with DPBS (ThermoFisher #14190250), BSC-GM was removed, and FBS-free screening medium (B8 or B9, which is B8 + 0.8 g/L HSA) supplemented with protein isolates were added at the designated concentrations. The volume of protein isolates was held below 40% of total cell culture volume. On indicated days Presto Blue assay was performed. Protein isolates were supplemented with every medium exchange.

### 2.8 Presto blue assay

To measure the metabolic activity, which correlates with the number of cells, Presto Blue assay was performed. Experiments were performed in 96-well plate format according to the instruction manual. Shortly, 10 μL Presto Blue reagent (ThermoFisher #A13262) was added to the remaining 90 μL of medium in each well before incubation at 37°C for 1 h. After incubation, the medium was transferred to a fresh 96-well plate (Greiner #655101) and the fluorescence at 560 nm (excitation) and 590 nm (emission) was measured with a plate reader (BioTek SynergyMx).

### 2.9 Confluency assay

For calculation of confluency, cells were seeded and treated the same way as described above for the short term proliferation experiments, but using Corning glass-bottom imaging plates (#CLS4580). For imaging, following parameters were used: exposure 50 ms, brightfield, Objective Plan Apo *λ* 4x, capturing using Andor Zyla VSC-08691NIS Elements. The images measure a size of 3113.3 μm x 3110.03 μm resulting in an area of 9,682,486 μm^2^. The values measured by the program are also given in μm and therefore can be expressed as a percentage in relation to each other.

### 2.10 Statistical analysis

For statistical analysis the data was tested for significance using ordinary one-way ANOVA followed by Tukey’s multiple comparisons test, using GraphPad Prism version 10.2.3. Statistical significance was reached when *p* < 0.05 (*), *p* < 0.01 (**), *p* < 0.001 (***) and *p* < 0.0001 (****). Non-significant results (*p* ≥ 0.05) were marked as “n.s.” The error bars in the graphs represent the standard deviation.

## 3 Results

### 3.1 Pumpkin and rapeseed protein isolates can induce higher BSCs proliferation

**Figure 1:**
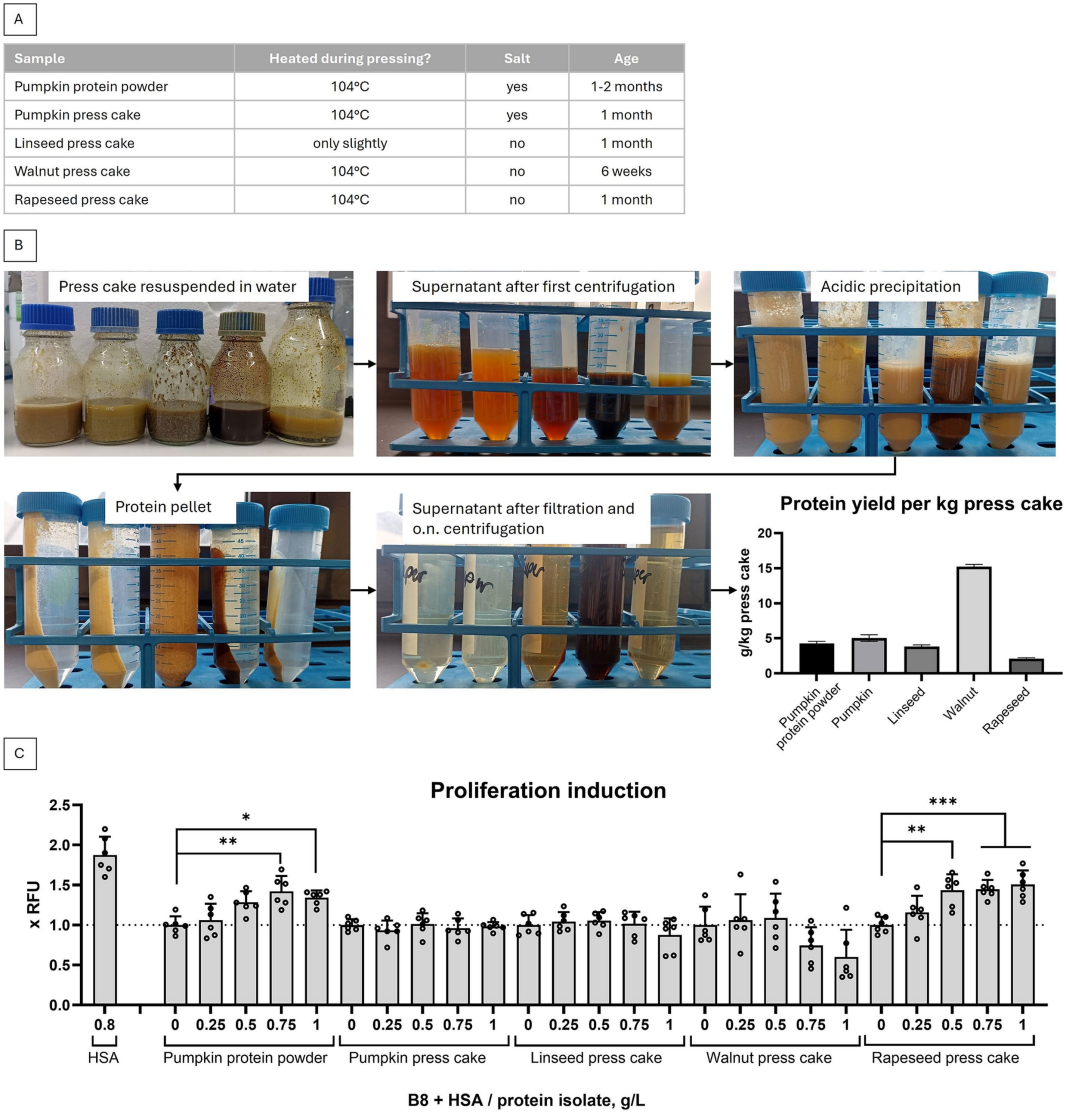
Non-optimized protein isolations from 5 press cakes. **(A)** The treatment of material in the process of press cake production is listed. **(B)** Protein isolates were produced following protocol from Stout et al. (9) from (left to right) pumpkin protein powder (produced by grinding pumpkin press cake), pumpkin press cake, linseed press cake, walnut press cake, rapeseed press cake. **(C)** Capability of press cake isolates to substitute HSA in B9 medium (B9 = B8 + 0.8 g/L HSA) in a short term proliferation experiment with BSCs. Presto Blue assay was performed on day 4. Obtained values were normalized to B8 medium. Sample size *n* = 6 and repeated twice; statistical significance was calculated by one-way ANOVA combined with Tukey’s test, comparing all samples to B8, and is indicated by asterisks, which are *p* < 0.05 (*), *p* < 0.01 (**), *p* < 0.001 (***), *p* < 0.0001 (****).

Five press cake preparations, i.e. pumpkin protein powder – fine grounded press cake and four coarsely grounded press cakes (pumpkin, linseed, walnut and rapeseed), were locally sourced from Ölmühle Lugitsch and information about their treatment in the process of press cake production was collected, see Figure 1A. All five were used to isolate protein fraction following previously published alkali extraction method (9). In the first isolation round, a coffee grinder was used to process the press cakes, as described. Pumpkin protein powder was also processed in the coffee grinder. Generally, the protocol was strictly followed, except that the reported concentration factor (50x to 100x) could not be reached. Instead, the maximum concentration factor of 7.25x was achieved. As shown in Figure 1B, the highest yield of protein per kg press cake was isolated from walnut press cake, although the concentrations were generally lower than the previously reported 40–200 g/kg of protein meal (9).

We have further tested press cake isolates for proliferation enhancing activity in a standard short term proliferation experiment using primary BSCs. We have observed that at least two protein isolates – from pumpkin and rapeseed – were able to induce higher proliferation rates, as compared to non-supplemented FBS-free, fully chemically defined B8 medium (Figure 1C).

### 3.2 Grinding efficiency defines the protein yield

The coffee grinder was pushed to its limits efficiently grinding certain pieces of press cake and generated significant heat during the process, which could negatively influence the integrity of the protein isolation and their biological activity. Thus, for the second isolation round we have applied a cryogenic grinder Pulverisette 2 (Fritsch, Cat# 02.2000.00) with liquid nitrogen for cooling to grind the press cakes. As only rapeseed and pumpkin isolates were demonstrating a proliferation inducing activity after the first isolation, we have chosen to work with these.

**Figure 2:**
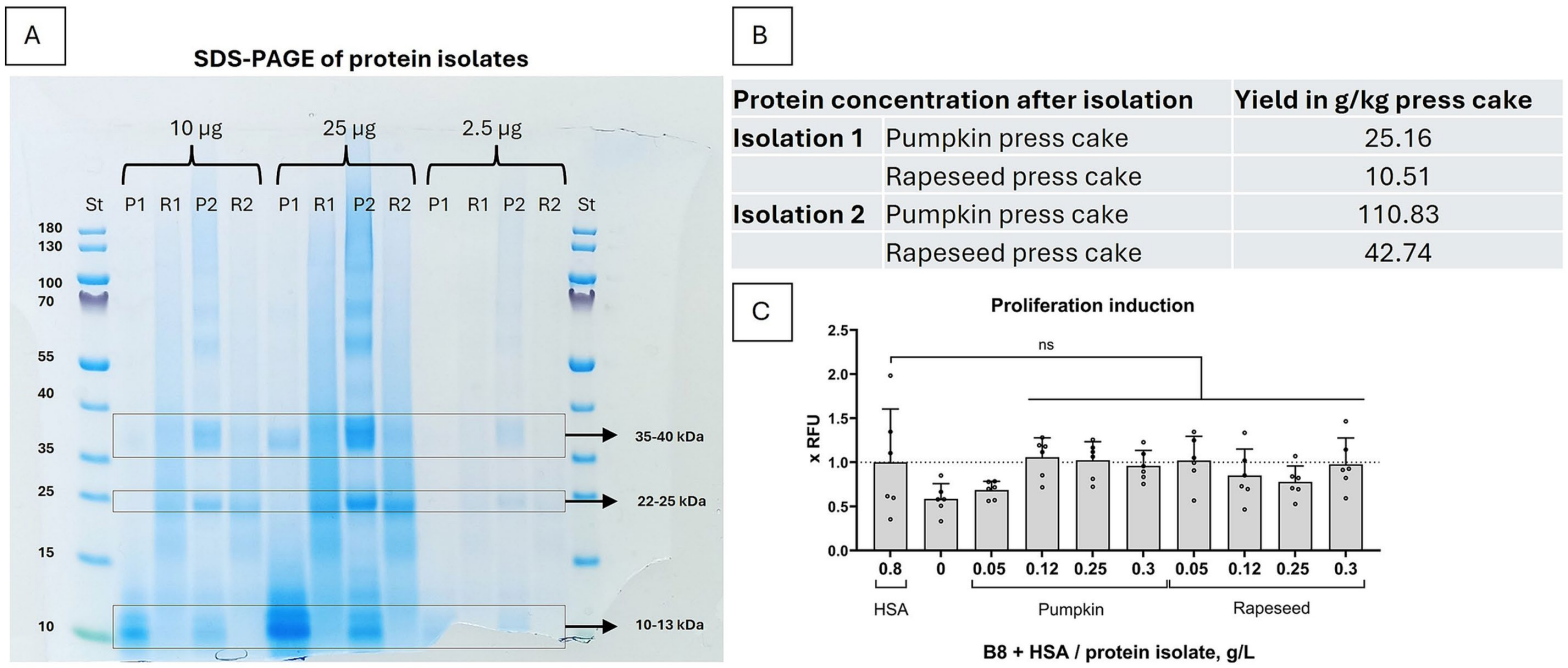
Protein isolation with higher degree of cooled particle grinding results in a significantly higher yield of plant proteins. **(A)** SDS-PAGE of the first (1) and second (2) protein isolations of pumpkin (P) and rapeseed (R). 2.5 μg, 10 μg and 25 μg total protein were loaded from first and second isolations. Enriched fractions were in the range of 35–40 kDa, 22–25 kDa and 10–13 kDa. **(B)** The upgrade of the grinding method in isolation 2 resulted in an overall 4x higher protein yield for both pumpkin and rapeseed press cake. **(C)** Biological activity of the second protein isolation, concentrated samples used. 3 to 10 times lower press cake isolates concentrations were necessary to substitute HSA in B9 medium (B9 = B8 + 0.8 g/L HSA) in a short-term proliferation experiment with BSCs. Presto Blue assay was performed on day 4. Obtained values were normalized to B9 medium. Sample size n = 6 and repeated twice; statistical significance was calculated by one-way ANOVA combined with Tukey’s test, comparing samples to B9, and is indicated by asterisks, which are p < 0.05 (*), *p* < 0.01 (**), *p* < 0.001 (***), *p* < 0.0001 (****).

We show that only by applying a more thorough and cooled grinding method the isolation yielded four times higher protein concentrations than previously (Figure 2B). Also, the SDS gel with the same protein amounts loaded for both isolations (Figure 2A) shows that the fractions corresponding to the plant albumins (around MW = 12–14 kDa), are not enriched in the second isolation. The band corresponding to pumpkin Cuc ma 5 (= 2S albumin), at MW = 12 kDa (12, 13) is much fainter in the second isolation, whereas bands corresponding to rapeseed Napins (= 2S albumins), with MW = 12.5–14.5 kDa (14, 15), are about of the same intensity. Instead, for pumpkin, the bands at 22–25 and 35–40 kDa are much stronger in the second isolation, which do not correspond to the known pumpkin albumins. For rapeseed isolates, the smear is significantly reduced in the second isolation, probably indicating a lower protein degradation level.

We have further tested the second isolation for biological activity in a short-term proliferation experiment. We demonstrated, that a much lower pumpkin and rapeseed protein isolate concentration was necessary to elicit the same proliferation inducing effect – 0.12 g/L of the second isolation, even 0.05 g/L in case of rapeseed (Figure 2C), as compared to 0.75–1 g/L in the first isolation (Figure 1C). The effect was also more comparable with B9 than in the proliferation experiment after the first isolation, which could be an indication that bigger proteins are eliciting an additional positive effect.

### 3.3 High concentration is not necessary for preservation of biological activity

We have further compared most widely applicable storage methods, alternative to storage at -80°C – lyophilization and spray drying. Ultrafiltration is an expensive step, which could probably be avoided if lyophilization or spray drying would provide storage options. Thus, additionally to concentrated samples, we also subjected non-concentrated samples to lyophilization and spray drying.

**Figure 3:**
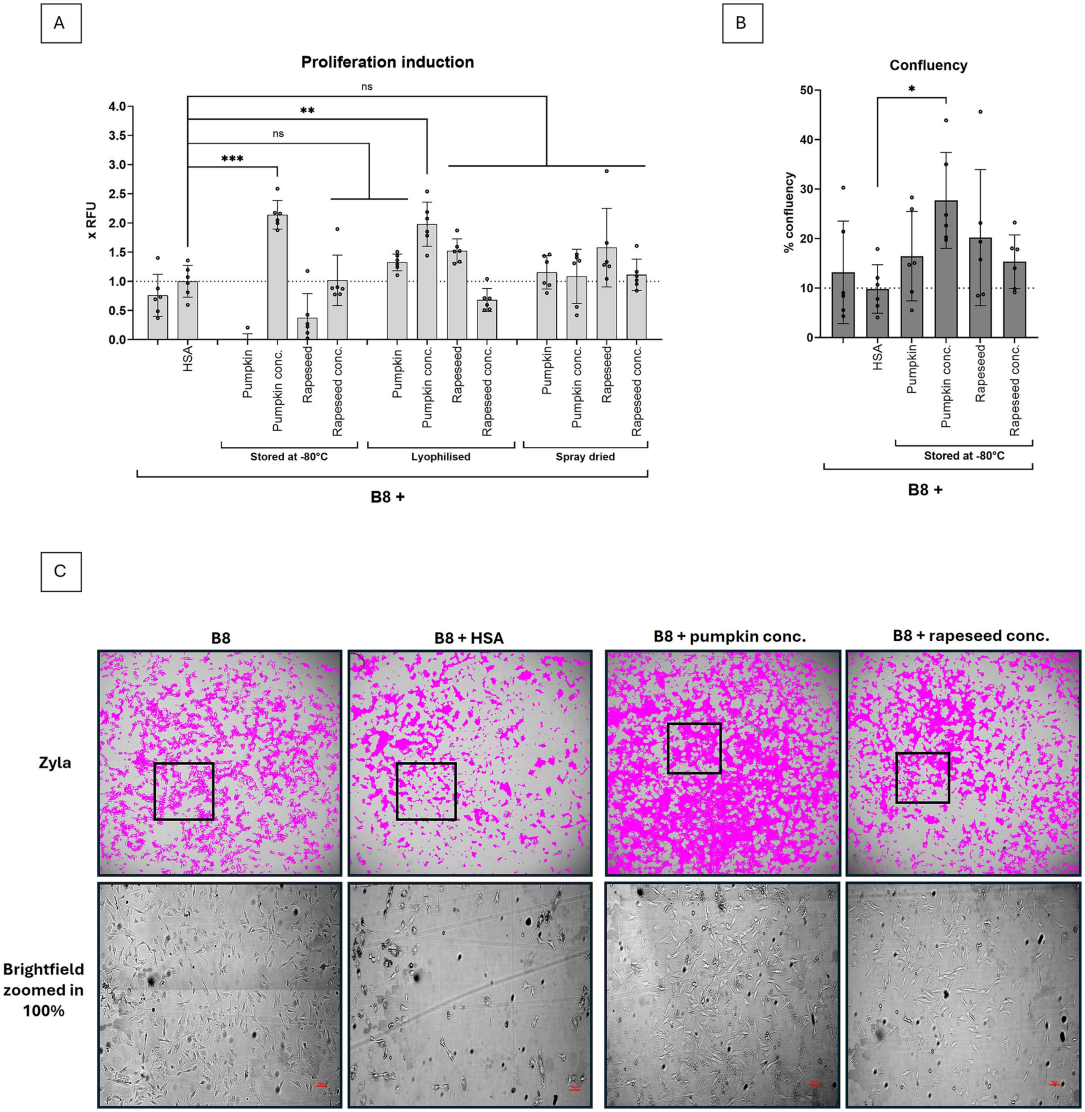
Effect of concentration and drying methods on biological activity of protein isolates. Pumpkin and rapeseed press cake isolates before and after concentration were either stored at -80°C, lyophilized or spray dried. **(A)** Biological activity of 0.12 g/L protein isolate was evaluated as a function of BSCs proliferation, measured by Presto Blue assay on day 4, and normalized to proliferation in B9 (B9 = B8 + 0.8 g/L HSA). Testing different protein isolates demonstrated that biological activity was strongly dependent on the storage method. Both non-concentrated protein isolates tolerated lyophilization and spray drying, but not the storage at -80°C. **(B)** Confluence measurements on day 4 were conducted to confirm Presto Blue data, and to ensure the cells are not under contract inhibition conditions. Sample size *n* = 6 and repeated twice; statistical significance was calculated by one-way ANOVA combined with Tukey’s test, comparing the mean of each condition with the mean of every other condition, and is indicated by asterisks, which are p < 0.05 (*), *p* < 0.01 (**), *p* < 0.001 (***), *p* < 0.0001 (****). Whole well images of the BSCs treated with protein isolates were taken to **(C)** assess confluence and morphological differences upon treatment. Images of day 4 are shown, scale bar = 100 μm. No morphological differences were found between B8 and B8 + protein isolates.

The samples were lyophilized or spray dried and stored at -20°C until their biological activity was investigated in a short-term proliferation experiment with 0.12 g/L protein end concentration. We show that rapeseed and pumpkin isolates show diverging tolerance towards different storage methods (Figure 3A). We confirm that the concentrated rapeseed isolate does not tolerate lyophilization, which is in line with previous reports (10). But non-concentrated rapeseed isolates tolerate lyophilization very well, as well as spray drying. Concentrated pumpkin isolate performed best when stored at -80°C (*p* < 0.001) or lyophilized (*p* < 0.01), but also non-concentrated lyophilized and spray dried isolates were performing better than B9 medium and comparable with rapeseed isolates, as measured by Presto Blue assay (Figure 3A). Generally, spray dried isolates performed slightly worse than lyophilized – which was expected – and for both isolates, alternative storage methods at -80°C were identified.

In an additional experiment, we confirmed that the Presto Blue data were obtained under non-contact inhibition conditions with confluence of less than 50% (Figure 3B). The confluence experiment partially supports Presto Blue data – at least in terms of the superior performance of the concentrated pumpkin isolate, but in contrast to the Presto Blue data also demonstrates acceptable performance of the non-concentrated pumpkin and rapeseed isolates compared to B8 or B9. This could probably be explained by the difference in adherence capacity of the cells to plastic (in the Presto Blue experiment) and to glass surfaces (in the confluence experiment), and could also be influenced by the albumins themselves, as shown in Stout et al. (3). The images, taken during the confluence experiment also show no morphological changes upon treatment with protein isolates, as compared to B8 control (Figure 3C), whereas bigger differences are detected between B8 and B8 + HSA controls, which is in line with previous reports (3).

## 4 Discussion

The use of albumins to stabilize cell culture media is a widely recognized practice, particularly in the cultivated meat sector (2). Notably, three out of five published serum-free (SF) media formulations incorporate either human serum albumin or bovine serum albumin combined with fetuin, both of which facilitate the proliferation of muscle stem cells (3–5). Thus, the production of serum albumins is expected to become a critical bottleneck for the cultivated meat industry, underscoring the urgent need to explore alternative solutions.

Recently, in-house produced plant protein isolates from press cakes – especially from rapeseed – enriched with plant albumins were proposed as alternatives to HSA (9). These alternatives are effective, low-cost, scalable, safe for food, and sustainable, with the input cost of rapeseed protein meal to make enough protein isolate for one liter of SF medium only $0.002 (9). Press cake protein isolate production is scalable, relying on only simple and low-cost alkali/acid extraction and filtration steps. It was shown to exceed recombinant albumin in promoting BSC growth while maintaining myogenicity (9). However, these isolates necessitate high concentrations and storage at -80°C to preserve their biological activity, while long-term storage at 4°C or lyophilization result in aggregation and a loss of biological efficacy (9).

In this study, we present one more press cake source - Styrian oil pumpkin – which can replace human serum albumin (HSA) as well as rapeseed protein isolates, in some cases even slightly better, depending on the storage method used. Furthermore, we show that the high protein yield upon isolation is highly dependent on the grinding efficiency of the press cake (Figure 2B). Also, the protein profile of the isolation – especially of the pumpkin isolate – has changed upon more efficient grinding, showcasing stronger protein bands, which do not correspond to the known plant albumins (Figure 2A). The proliferation-inducing effect observed in the second isolation was significantly greater than that of the first isolation, with a lower protein concentration required (Figures 1C, 2C). This difference may be attributed to a greater proliferation-promoting effect of the unidentified, enriched proteins. Future research should focus on identifying these proteins and further optimizing the isolation protocol to enhance their enrichment.

Following the second isolation, a minor decrease in the proliferation rate was observed at protein concentrations exceeding 0.12 g/L (Figure 2C). In contrast, the initial isolates exhibited an increase in proliferation induction up to 1 g/L of protein (Figure 1C). This observation may suggest the possibility of co-isolation or enrichment of potentially toxic components due to more efficient grinding. Subsequent research should thoroughly investigate this possibility by conducting an exhaustive analysis of the protein isolates’ composition.

We also demonstrate that protein isolates derived from pumpkin and rapeseed press cakes can be lyophilized or spray-dried without significant loss of activity, although spray drying is somewhat less favorable. This is contingent upon the omission of the concentration step. These findings not only reduce the necessity for storage at -80°C, thereby lowering storage costs, but also render the ultrafiltration step unnecessary, which requires specialized equipment and incurs additional energy costs.

Based on the results of this study, we provide a more user-friendly, sustainable, and locally sourced alternative to human serum albumin (HSA) for SF media formulations, along with a more price-efficient storage methods thereof.

## Data Availability

The raw data supporting the conclusions of this article will be made available by the authors, without undue reservation.
